# Amniotic Membrane as a Scaffold for Melanocyte Transplantation in Patients with Stable Vitiligo

**DOI:** 10.1155/2011/532139

**Published:** 2011-08-18

**Authors:** Pedro Redondo, Ana Giménez de Azcarate, Laura Marqués, María García-Guzman, Enrique Andreu, Felipe Prósper

**Affiliations:** ^1^Department of Dermatology, University Clinic of Navarra, University of Navarra, 31008 Pamplona, Spain; ^2^Area of Cell Therapy, University Clinic of Navarra, University of Navarra, 31008 Pamplona, Spain

## Abstract

Vitiligo is an acquired skin disease that significantly impacts the quality of life of patients. Medical treatment of vitiligo includes the use of melanocyte transplant, but the results are variable. We have treated 4 patients with either focal or generalized stable vitiligo using a graft of autologous melanocytes' culture on a denuded amniotic membrane (AM). A culture biopsy was obtained in every patient and grown in melanocytes' media for 10–14 days after which cells were transferred to a denuded AM and transplanted into the achromic lesions. Patients were followed for up to 6 months using clinical assessment of achromic lesions. Treated areas ranged between 4 cm^2^ and 210.6 cm^2^. Response to treatment was excellent in all patients with 90–95% repigmentation success rate. Our results demonstrate that transplantation of autologous melanocytes cultured on AM is a new, simple, and effective treatment for stable vitiligo.

## 1. Introduction

Vitiligo is an acquired skin disease that affects 0.1–3% of the world's population, characterized by loss of melanocytes from the epidermis, and leads to the development of achromic lesions. The basic pathogenesis of vitiligo remains unknown, although several studies suggest genetic predisposition, relationship to other autoimmune disorders, biochemical and neurohormonal imbalance, and environmental toxin/stressors [1–3]. The cosmetic disfigurement caused by vitiligo has profound psychological effects in approximately two-thirds of the patients, with depression, low self-esteem, social rejection, and even job discrimination [4]. In patients affected by focal vitiligo, the causative factors usually disappear, leaving well-defined achromic lesions. In stable generalized vitiligo, the size and number of lesions are stationary for several years and the Koebner phenomenon is absent. Current conventional medical treatment of vitiligo consists of UV therapy (narrowband UV-B or psoralen plus UV-A), local steroids, tacrolimus, pimecrolimus, and calcipotriol. In patients with stable vitiligo, lack of reliably effective medical therapies has led to the development of surgical treatment options using transplantation of autologous melanocytes. This technique includes split-thickness grafts, punch grafts, and suction blister grafts, which do not require cell expansion [5, 6]. Complications of these surgical methods can lead to the appearance of a cobblestone surface, peripheral hypopigmentation, spotty pigmentation, or lack of pigmentation of the treated areas, as well as to scarring and pigmentary alterations of the donor sites [7]. Currently transplantation of cultured autologous melanocytes with or without keratinocytes for treating vitiligo is at the developmental stage. Such transplantation techniques include noncultured epidermal suspension, cultured mixed melanocyte-keratinocyte suspension with or without carrier, and cultured pure melanocyte suspension [8, 9].

The amniotic membrane (AM), the inner part of the placenta, consists of a thick basement membrane of collagen type IV and laminin and an avascular stroma. Davis [10] was the first to use fetal membranes in skin transplantation in 1910. Subsequently, AM underwent widespread use as biological dressing in the management of burns [11], chronic venous leg ulcers [12], toxic epidermal necrolysis [13], epidermolysis bullosa [14], surgical dressings [15], and following facial dermabrasion [16]. Since 1995, when Kim and Tseng reported the use of preserved human AM to rehabilitate severely damaged rabbit cornea [17], AM transplantation has been successfully applied for ocular surface reconstruction in patients with severe corneal diseases [18].

Experimental and clinical studies using AM as a graft or patch have demonstrated that AM promotes re-epithelialization, decreases inflammation and fibrosis, and inhibits angiogenesis. AM acts like a basement membrane and facilitates the migration of epithelial cells [19], has an anti-inflammatory effect by inhibiting protease activity and infiltration of leukocytes and by suppressing IL-1*α* and IL-1*β* [20], and induces downregulation of TGF-*β* thus reducing fibrosis [21]. In addition, AM has antimicrobial properties that decrease the risk of postoperative infection [22]. Moreover, AM has been thought to display very low immunogenicity. The technique of human AM processing and cryopreservation with the Dulbecco Modified Eagle Medium and 50% glycerol recommended by the FDA renders all the amniotic cells nonviable [23].

In the current study, we report the clinical results obtained with the application of melanocytes cultivated on AM onto distinct achromic lesions.

## 2. Patients and Methods

### 2.1. Patients

The study was approved by the institutional ethics committee, and written informed consent was obtained from the patients or parents. From January 2005 to May 2006, 4 patients (1 male and 3 female) ranging in age from 13 to 29 years, 2 with stable generalized vitiligo and 2 with stable focal vitiligo were treated with autologous transplantation of pure melanocytes using AM as a carrier (Table 1). Stable disease was defined as no new lesions or expansion of preexisting lesions in the last 12 months. All patients had previously received several medical treatments without response.

### 2.2. Amniotic Membrane

**Figure 1:**
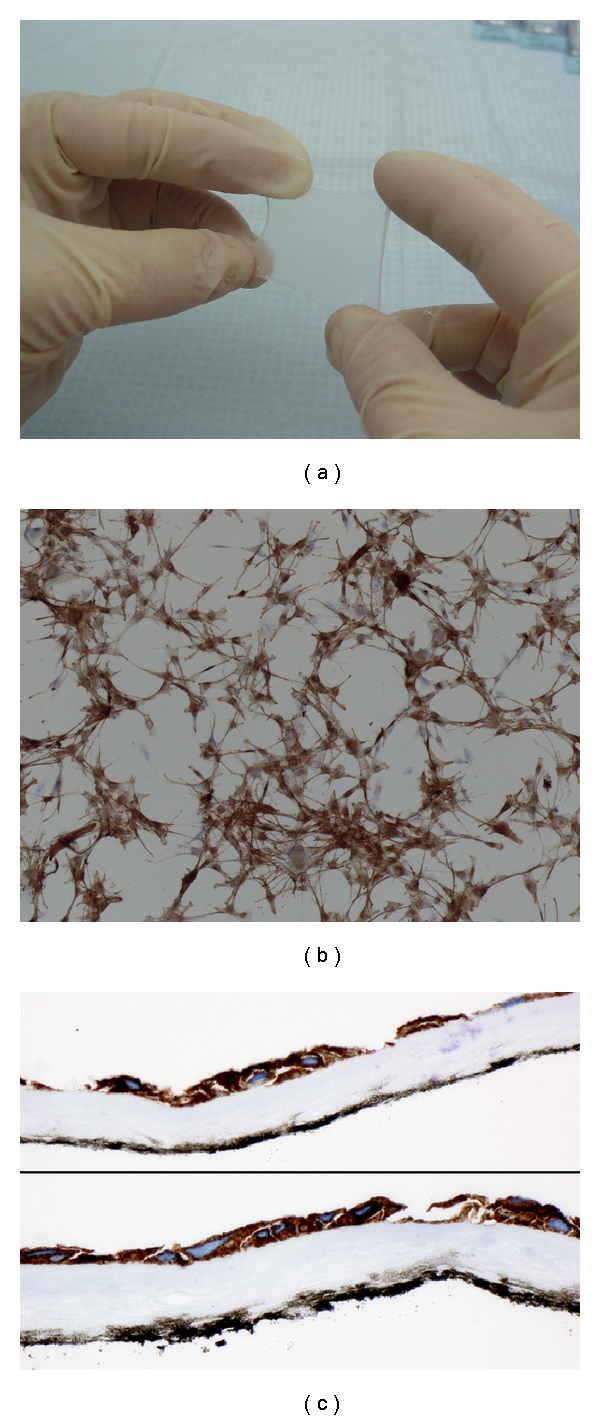
Piece of amniotic membrane (a); immunohistochemistry for HMB-45 applied to cells cultured on slide. A large number of melanocytes can be observed (b); micrograph of cross-sectioned AM stained with HMB-45 after cell culture (c). Original magnification: (b, c) X100.

Placentas were obtained during elective cesarean delivery from mothers who had been screened for HIV, hepatitis B, hepatitis C, HTLV-1, CMV, and syphilis at the time of birth, and 3 months after harvesting the AMs. Under a laminar flow hood, the placenta was washed free of all blood clots using balanced saline solution containing 50 ug/mL streptomycin, 50 ug/mL penicillin, and 2.5 ug/mL amphotericin. The amnion was separated from the rest of the chorion by blunt dissection. The membrane was then flattened onto a nitrocellulose paper, with the epithelium/basement membrane surface up. The membrane and the paper were then cut up into 5 × 5 cm pieces and placed in a sterile vial containing the Dulbecco Modified Eagle Medium and glycerol in a ratio 1 : 1. The vials were frozen at −80°C. Immediately before use, AM was thawed and washed with PBS. The pieces were then treated with 0.02% ethylenediamine tetraacetic acid (EDTA) in PBS at 37°C for 2 h. The digested AM was gently scrubbed with a plastic spatula to remove the epithelium without breaking the basement membrane. Acellularity of the scrubbed AM was confirmed by H&E staining (Figure 1(a)). AM was placed in a plastic frame before seeding of melanocytes.

### 2.3. Cell Culture

Melanocyte basal medium (MBM, PromoCell, Heidelberg, Germany) without PMA (phorbol 12-myristate 13-acetate) nor bovine pituitary extract, supplemented with basic fibroblast growth factor (1 ng/mL), human recombinant stem cell factor (50 ng/mL), and human recombinant granulocyte/macrophage colony-stimulating factor (10 ng/mL) for 10–14 days, was used for the melanocyte culture. 

A superficial shave biopsy (1 cm^2^) was taken from pigmented buttock skin under local anesthesia. Skin samples were washed in phosphate-buffered saline (PBS), and the connective tissue was trimmed. The epidermis was separated from the dermis with 1% Dispase II (Boehringer Mannheim, Barcelona, Spain) in PBS with gentamicin (20 mg/mL) at 37°C overnight. Epidermal sheets were peeled from the dermis and stirred in 0.05% trypsin and 0.53 mM EDTA solution (trypsin-EDTA solution) for 10 min at 37°C. The trypsin-EDTA solution was inactivated with a trypsin inhibitor (Sigma, Madrid, Spain) and centrifuged at 1500 rpm for 5 min. Cell pellets were resuspended in 10 mL of supplemented MBM and cultured at 37°C in a humidified atmosphere containing 5% CO_2_ in 25 cm^2^ tissue culture flasks (Nunclon, Roskilde, Denmark). When 80–90% confluence was reached (Figure 1(b)), cells were digested with 0.25% trypsin/0.02% EDTA and then replated into the basement membrane side of AM prepared as described above at a density of 5–25 × 10^3^ cell/cm^2^. Melanocytes were cultured for an additional 3-4 days and either transplanted onto the denuded skin or fixed with formalin for immunohistochemical staining.

### 2.4. Immunohistochemical Studies

To ensure the presence of melanocytes, immunohistochemical staining for HMB-45 (1 : 100; Biogenex, San Ramon, Calif, USA) was performed using the EnVision+ System (Dako, Glostrup, Denmark) according to the manufacture's recommendations. Both AMs and cells cultured on chamber slides were fixed in 4% neutral buffered formalin for 10 min. After washing in PBS, free-floating amniotic membranes and slides were permeabilized with 5% Triton X-100 for 30 min and endogenous peroxidase activity was quenched with 3% H_2_O_2_ for 10 min. Samples were incubated with primary antibody overnight at 4°C. After rinsing, membranes and slides were incubated with goat antimouse (K4001, Dako) labeled polymer for 30 min at room temperature. Cells were visualized by incubating the sections with DAB+ (K3468, Dako). Washes between each step were carried out with Tris-HCl 0.05 M buffer, 0.5 M saline, pH 7.6 (TBS) containing 0.05% Tween 20 (TBS-T). Slides were counterstained lightly with hematoxylin, dehydrated in graded series of ethanol, cleared in xylene, and mounted in DPX. Amniotic membranes were mounted onto glass slides, dried overnight, cleared in xylene, and coverslipped with DPX. Samples were examined on a Nikon Eclipse E800M microscope, and 10 random images per slide were captured with Analysis Soft Imaging System Gmbh software. The number of melanocytes per mm^2^ was counted using the ImageJ software.

### 2.5. Surgical Procedure

After disinfection with povidone-iodine, under local anesthesia with lidocaine hydrochloride (20 mg/mL), epidermal ablation was performed using the Silk Touch Flashscanner attached to a Sharplan 1030 CO_2_ laser at the setting of 5.5–7 W with a 0.2-second pulse duration (Laser Industries Ltd, Tel Aviv, Israel). To obtain an adequate depth of the wound bed in the deepithelialization of achromic areas, we ablated slightly less tissue than was necessary to achieve pinpoint bleeding, which indicates penetration into the papillary dermis. One laser pass was sufficient for all patients. After the papillary dermis was reached, the denuded lesions were treated with the AMs containing cultured melanocytes. AMs with the melanocytes were mounted on Vaseline gauze (LINITUL, Bama-Geve, Barcelona, Spain). Each amniotic membrane (surface 12.56 cm^2^) was secured, with the basement membrane with cultured melanocytes down, onto the denuded surface with one layer of Vaseline gauze, steri-strips, and several layers of dry gauze and traditional bandages.

Patients were asked to limit movement of the treated region for 3-4 days. At that time the bandage was removed. Two weeks after the procedure, all patients began to receive sun exposure or UVA irradiation twice per week for approximately 6 weeks to stimulate melanocyte proliferation.

## 3. Results

### 3.1. Cell Cultures' Analysis

The presence of melanocytes in the culture was confirmed on the basis of the morphology of the cells and immunocytochemical analysis with HMB-45. The number of melanocytes transplanted varied between 0.1 and 0.3 × 10^6^ per membrane. When melanocytes were transferred to AM, a parallel culture on chamber slides was also performed, and the percentage of melanocytes was greater than 90% in every case.

### 3.2. Clinical Outcome

**Figure 2:**
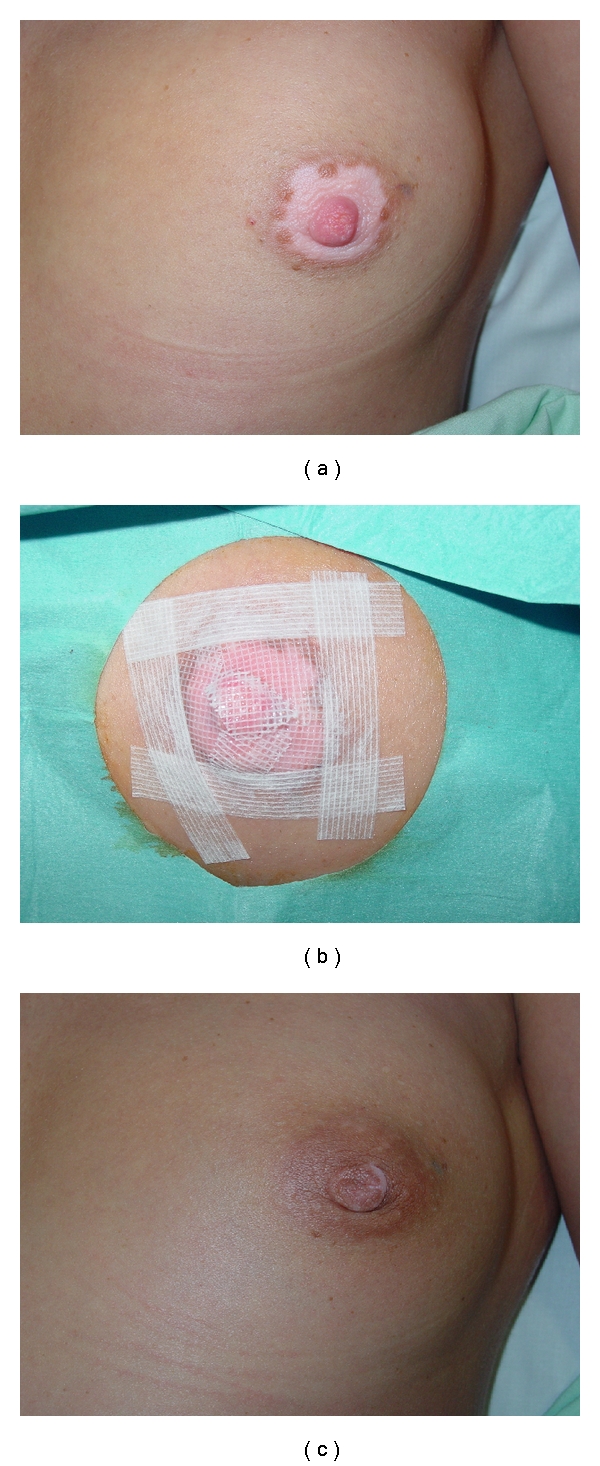
Patient 1. Preoperatively (a); the achromic epidermis was removed using CO_2_ laser, and AM carrying cultured melanocytes on the basement membrane surface was applied on the denuded area with one layer of Vaseline gauze. The amniotic membranes were fixed with steri-strips and covered with dry gauze and adhesive tape (b); postoperative photography (>90% repigmentation at 16 weeks) (c).

Achromic epidermis was removed by means of the CO_2_ laser. The largest area treated in one session was 210 cm^2^, and the smallest treated lesion was 4 cm^2^. Assessment of repigmentation was performed monthly for up to 6 months in every patient. While responses were observed after 2 months in some patients, the complete evaluation of the percentage of repigmentation in all patients was performed after 6 months (Figure 2). 

**Figure 3:**
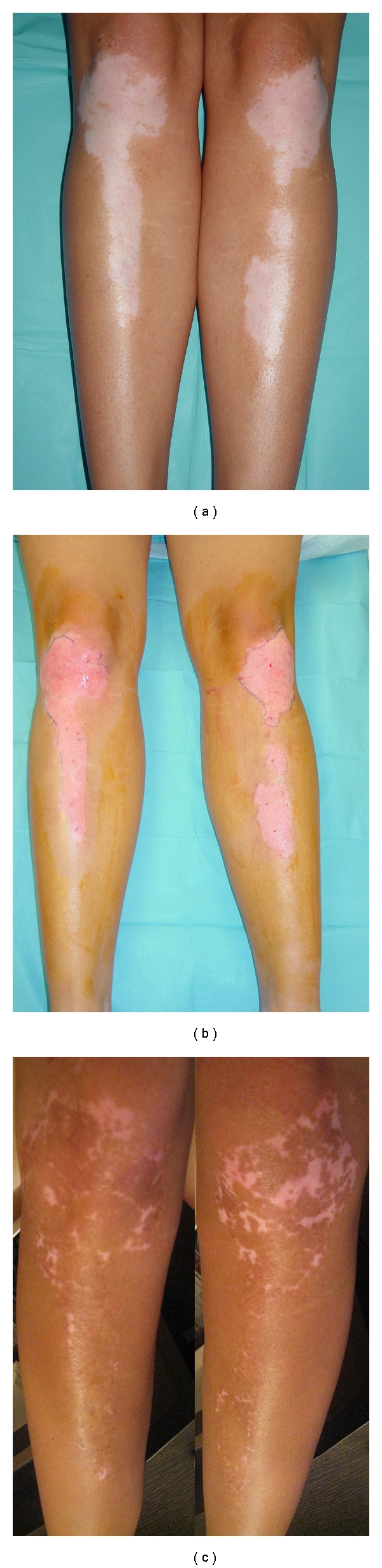
Patient 3. Preoperatively (a), desepidermized lesion after treatment with CO_2_ laser (b), and postoperative photographs (>90% repigmentation at 16 weeks) (c).

Response to treatment was excellent in all patients (Table 1). There were no differences in repigmentation according to the concentration of melanocytes in the graft. Interestingly, in all patients the marginal area of the hypopigmented lesions was also repigmented, unlike what is observed in other surgical procedures, where the edge of the lesions remains depleted of melanocytes. On the other hand, epidermis regeneration was completed 6 days after grafting, and no scars were observed in the recipients' areas (Figure 3).

## 4. Discussion

Despite the availability of various types of medical and surgical treatments for vitiligo, the therapeutic response of either focal or chronic stable generalized vitiligo to conventional treatment is poor, with a significant number of patients failing to respond with a satisfactory degree of repigmentation [24]. Cellular grafting techniques have advantages over tissue grafts such as punch grafts or suction blister epidermal grafts, including greater uniformity of pigmentation, lower risk of skin-texture abnormalities such as cobblestoning, and the ability to cover larger areas of vitiligo with smaller graft sizes. Noncultured epidermal suspension transplantation in stable vitiligo was first described by Gauthier and Surleve-Bazeille [25], and a recent paper reports the use of this technique in children and adolescents [26]. However, the donor skin size to recipient skin size ratio is between 1 : 3 and 1 : 10. Expansion of melanocytes in vitro provides more cells for transplantation; therefore, the cells from a small piece of skin can be used to treat a large vitiliginous area. One of the limitations of transplantation of cultured melanocytes is the delivery of the cells in a vehicle that facilitates the cells to engraft and remain in the location of the skin lesion. Administration of cell suspensions may lead to the disappearance of melanocytes while the use of different scaffolds may provide a better substrate for engraftment. Recently we treated a group of five patients with either focal or generalized stable vitiligo using a graft of autologous melanocytes cultured on a denuded AM, with very good repigmentation rates (90–100%) [27]. Here we treated two patients with vitiligo lesions greater than 200 cm^2^ with similar satisfactory results. From the second week the proliferation of melanocytes is exponential so that large achromic areas have no direct therapeutic limitation. The use of AM as a mean of providing a scaffold for transplantation of melanocytes offers several advantages: the anti-inflammatory and antimicrobial properties of the AM along with the lack of antigenicity facilitate the epithelization of the skin and decrease scarring. The basement membrane and its underlying extracellular matrix of AM containing collagen type IV, type VII, and laminins-1 and -5 [28] provide a natural substrate for cells and allow adhesion and proliferation of melanocytes. For these reasons AM seems to be a very good substrate for cultivating human melanocytes similarly to what is observed with human limbal epithelial cells [29]. Besides their biological properties, AMs are easy to obtain and unexpensive, can be customized, and are cosmetically desirable, avoiding the scars seen with the use of split-thickness skin grafts.

It has been hypothesized that epidermal trauma (dermabrasion or laser) could be a melanocyte-stimulating trigger to a reservoir of melanocytes, that is, hair follicles and the surrounding skin. On the other hand, the induced repigmentation might be postinflammatory, caused by the release of inflammatory mediators after epidermal trauma. We believe these are unlikely explanations for the observed effects in our patients in accordance to studies in which Van Geel et al. [30] have shown that repigmentation induced by epidermal ablation, UV therapy, and inflammatory mediators in placebo-treated lesions is extremely limited.

We also show that the use of CO_2_ laser allows precise removal of the epidermis, preservation of the papillary dermis, and absence of bleeding and inflammation being a user-friendly procedure, fast and uniform unlike more classical dermabrasion or diathermosurgery [31]. 

The results in this limited number of patients warrant further studies to demonstrate the usefulness of the AM as a scaffold for melanocytes. Only an intraindividual clinical trial evaluating the usefulness of AM as a scaffold versus the application of melanocytes in suspension can confirm the greater efficiency of using a carrier for transporting and transplanting the cells.

## Figures and Tables

**Table 1:** 

Patients	Age, years/gender	Type of vitiligo	Grafted area	Size, cm^2^	% repigmentation at 6 months
1	29/F	Focal	Areola and nipple	4	95
2	13/M	Focal	Chin	9	90
3	26/F	Generalized	Legs	185	90
4	17/F	Generalized	Legs	210	95
